# Applications of Ultrasound-Mediated Drug Delivery and Gene Therapy

**DOI:** 10.3390/ijms222111491

**Published:** 2021-10-25

**Authors:** Juliana Sitta, Candace M. Howard

**Affiliations:** Department of Radiology, University of Mississippi Medical Center, Jackson, MS 39216, USA; jsitta@umc.edu

**Keywords:** gene therapy, cancer, metastasis, systemic targeting, viral vectors

## Abstract

Gene therapy has continuously evolved throughout the years since its first proposal to develop more specific and effective transfection, capable of treating a myriad of health conditions. Viral vectors are some of the most common and most efficient vehicles for gene transfer. However, the safe and effective delivery of gene therapy remains a major obstacle. Ultrasound contrast agents in the form of microbubbles have provided a unique solution to fulfill the need to shield the vectors from the host immune system and the need for site specific targeted therapy. Since the discovery of the biophysical and biological effects of microbubble sonification, multiple developments have been made to enhance its applicability in targeted drug delivery. The concurrent development of viral vectors and recent research on dual vector strategies have shown promising results. This review will explore the mechanisms and recent advancements in the knowledge of ultrasound-mediated microbubbles in targeting gene and drug therapy.

## 1. Introduction

Cancer is currently the second major cause of death in the United States (USA). Despite increasing research investment and new therapeutic developments, overall cancer death rates have improved only modestly in the past 20 years in the US, from 200.7 per 100,000 people in 1999 to 149.2 per 100,000 people in 2018 [1]. Moreover, death rates in specific types of cancer such as hepatobiliary, pancreatic, uterine, renal, laryngeal cancers, and multiple myeloma have worsened or shown no improvement whatsoever over this same period of time. A lack of improvement in death rates largely reflects the lack of major treatment advances for patients bearing metastatic or recurrent disease. Additionally, most of the treatment modalities available, particularly for advanced stages of cancer, commonly cause marked side effects, often resulting in high rates of withdrawal due to low patient tolerability [2,3].

Over recent years, the field of cancer therapy research has progressively focused efforts on new strategies to increase treatment efficacy while minimizing side effects. The emergence of gene therapeutic approaches to treat genetic abnormalities and cancer about 30 years ago, brought excitement and hope in the scientific community. However, translation of gene therapy concepts to clinical practice in the early 1990s was halted by disappointing results. Several of these clinical trials either were unable to show benefit or were withdrawn due to marked side effects and even widely publicized fatalities. Almost a decade later, there was new enthusiasm with a second generation of gene transfer vectors which demonstrated promising results in animals. However, progress was again slowed by the emergence of serious toxicities such as insertional genotoxicity, immune destruction of genetically modified cells, and immune reactions related to certain vectors [4,5].

The gene therapy concept relies on the transfer of genetic material to repair, regulate, or replace defective genes with a goal to treat a target disease. The gene therapy field has evolved from the treatment of single gene disorders to a broader spectrum of strategies including cell death induction and protein production [6]. Several studies have demonstrated the advantages of a multi-modality approach in various types of cancer, reporting the usefulness of gene therapy as either immunotherapeutic, radiotherapeutic or chemotherapeutic sensitizers or enhancers [7,8]. One of the most common challenges encountered by scientists over the years is the development of a suitable delivery system that allows introduction and stabilization of nucleic acids within the target cell. Since nucleic acids are rapidly cleared from the system by phagocytes or nucleases, a delivery system, also known as vectors, is required in order to allow nucleic acids to reach the nuclei of host cells and to induce gene expression. Thus, a successful gene transduction requires three basic components which includes a vector-based gene expression system that is able to induce function of a gene in a host cell, a gene that encodes a specific therapeutic protein, and a gene delivery system that controls the delivery of the gene expression vector to a specific location within the body [9].

The currently available gene vectors are classified as non-viral, viral, and hybrid. Non-viral physical or mechanical vectors usually include simple systems such as plasmid DNA, lipid, chromosomes, naked DNA, cationic polymers, and conjugate complexes. Mechanical strategies include the direct introduction of genetic material via electroporation, microinjection, magnetofection and gene-gun-based methods. The main advantage of non-viral vectors over viral vectors is the reduced host immunogenicity response. However, the current available non-viral transfection methods have shown marked lower efficacy compared to viral vectors [5].

Viral-based transfection consists of genetically engineered virus carrying specific nucleic acid sequences into a host cell. Viral-based transduction is classified as stable or transient transfection. In the former, upon entering the host cell, the viral genetic material is integrated into the host cell genome, and the new addition is continuously expressed through the subsequent cell progeny. In contrast, with transient infection, the viral genome is episomal, and stable integration is not guaranteed. Retroviruses such as lentivirus are commonly used to generate stable transfection, while adenoviruses, adeno-associated viruses, and herpes viruses are commonly used for transient infection. The main disadvantage of viral-based vectors is the increased host immunogenicity and risk of viral infection. Retroviruses typically exhibit lower rates of host inflammation than adenovirus and herpes virus; however, stable transfection is associated with a high risk of insertional mutagenesis and gene disruption, and retroviruses are limited by the ability to only infect dividing cells [5].

Given the advantages and disadvantages of non-viral and viral-based vectors, one of the main challenges in gene delivery has been the development of a delivery system that is consistently safe and efficacious for clinical application. A recently developed approach to overcome these obstacles and generate targeted and safe transfection with reduced risk of host immunogenicity is the use of an ultrasound-guided microbubbles (MBs) delivery system. This article explores the current developments and prospective research in targeted delivery of gene therapy using MBs ultrasound contrast agents.

## 2. MBs Mechanics and Ultrasound Technique

Ultrasound (US) is a nearly innocuous and widely available imaging technique with a well-established role in various diagnostic applications. Diagnostic US techniques uses high frequency ultrasound waves to view real-time tissue and organs inside the human body. The use of US as a drug delivery facilitator was first described in the mid 90s, using the physical transient increased cell membrane permeability from sonoporation [10,11,12]. Subsequent research reported the enhanced biophysical effects of ultrasound by incorporation of MBs [13,14].

The use of MBs as ultrasound contrast enhancement agents has dramatically evolved in recent years, particularly after the US Food and Drug Administration (FDA) first approval for use in clinical practice in 2001. MBs are gas-filled spherical voids coated by a stabilizing shell composed of phospholipids, proteins, or synthetic polymer materials, measuring approximately 1–10 um. The difference between the acoustic impedance of the MBs’ gas filling composition (e.g., perfluorocarbon, sulfur hexafluoride, or nitrogen) and the surrounding blood is highly reflective and generates an enhanced acoustic backscattering from blood [15].

In clinical practice, diagnostic US takes advantage of the physical properties of MBs made possible by its resonance behavior. Under the compressibility variations of US waves along with the surrounding liquid inertia, MBs respond with a mass-spring-like resonance behavior whose frequency obeys an inverse relation with the bubble radius [16]. Thus, at routinely diagnostic low acoustic powers, MBs compressibility mainly generates synchronous oscillations and linear echo emissions, which provides contrast enhancement commonly used in assessment of cardiac function and characterization of visceral lesions in diagnostic imaging [17].

However, under effects near its resonance frequency, the bubble displays maximal radial response and generates secondary effects, such as harmonics and subharmonics, microstreaming, acoustic radiation forces, shape instabilities, and non-spherical oscillations [16]. These effects have been previously described in clinical applications such as non-invasive vascular pressure estimation [17], bacterial biofilms removal [18], mechanical destruction of thrombus or tumors [19], or vessel wall permeability induction [20].

Given MBs’ compressible core, they are able to respond to ultrasound pressure wave oscillations, a process known as cavitation. Thus, cavitation of MBs varies according to resonance frequency, pulse repetition frequency, acoustic power, gas core composition, damping coefficients, and shell properties [21]. Since acoustic power is the most important US parameter to determine MBs’ response, it further divides the cavitation process into stable, when low acoustic pressure ultrasound is applied, or inertial, when high acoustic pressures are applied.

The cavitation process at low acoustic pressures is called stable, because the net of influx and efflux of gas during MBs’ compressibility and expansion phases is zero. At high acoustic pressures, however, the expansion phase is extended and MBs oscillate in a non-linear fashion.

Furthermore, when expansion reaches its resonant size, MBs oscillate in a low amplitude, creating microstreams of blood flow around them [22]. Coupled with microstreams, the ultrasound acoustic radiance force generates displacement of fluid and particles in the direction of the sound wave propagation driven by the radiance force from scatters and reflectors in the ultrasound field, a process known as bulk stream. The bulk stream is important in gene therapy applications, since distance between MBs and the target cell membrane has been observed to influence the degree of pore formation in previous studies [23,24]. The intensity of the bulk and micro streams is dependent on the applied US parameters, and when in proximity to the blood vessel wall, is able to produce shear stress, inducing pore formation [25,26,27]. Importantly, the biological effects of the microstreaming production vary drastically according to the acoustic pressure setting. Specifically, at lower acoustic pressures MBs micro shearing induces rapidly reversible pore formation; while at higher acoustic pressures, pore formation may be followed by cell death. Each setting may be applicable in different clinical scenarios. For instance, the reversible pore formation has been used to safely transpose membranes and target drug and gene therapy to diseased tissue. On the other hand, higher acoustic pressures have been used when tissue death is desirable such as oncologic applications [20].

Previous studies hypothesized that the cavitation process in MBs interacts with the cell membrane integrity by generation of a push and pulling effect. Multiple authors studied the impact of different levels of acoustic pressure on the cellular membrane deformation. Experimental studies demonstrated a high correlation between acoustic pressure level and cellular membrane deformation. Wang et al. also studied the impact of bubble-to-cell distance and their interaction using a boundary element method model. The authors found that the degree of cell membrane deformation inversely correlated with widening of the bubble–cell membrane gap [28].

Besides the effect of acoustic radiation force, another potentially useful approach to reduce the bubble-to-cell membrane distance is the use of targeted MBs. Kooiman et al. investigated the influence of microbubble targeting in sonoporation effectiveness and reported that targeted MBs are able to induce pore formation at peak negative pressures 2–5 times lower compared to non-targeted MBs [7].

**Figure 1:**
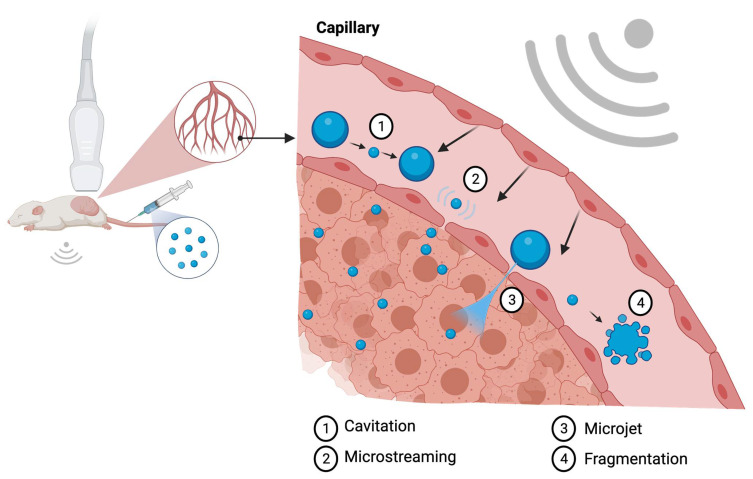
Illustration of the biologic effects of MBs sonification at the capillary level. The cavitation process (1) represents the change in MB diameter reflecting in expansion and shrinking resultant of acoustic pressure variation. Microstreaming (2) is regarded as one of the major biological effects able to induce pore formation through a process of microshearing. Microjet formation (3) and fragmentation (4) are the mechanisms observed at the maximal expansion phase of the cavitation process. Created with BioRender.com.

A second mechanism used by the ultrasound microbubble system for enhanced targeted therapy delivery is microjet formation. Once the extended expansion generated by high acoustic pressure leads to MBs collapse, a membrane disruption may be produced resulting in microjet formation. The microjets are generally oriented towards the shock wave propagation direction; however, when in proximity to highly reflective tissues, the direction of the microjet may be less predictable [29]. Endothelial cell membrane pore formation by shear stress generated by microstreams and microjet formation are the main mechanisms utilized to enhance tumor drug delivery, since it allows for increased localized vessel permeability. Figure 1 illustrates the biophysical effects of ultrasound on MBs.

Another important ultrasound parameter reported to modify cell membrane response to microbubble cavitation is pulse length. It is known that MBs move toward each other as a consequence of secondary acoustic radiation forces, which is increased by longer pulse lengths, and that increases aggregation rate, reducing delivery effectiveness. Experimental studies investigated the microbubble behavior at two different wave lengths: 10 ms and 10 μs, maintaining acoustic pressure at 400 kPa. The authors observed high delivery rates and higher cell viability with short pulse length and massive cell death with low delivery rates at longer pulse length [30]. Thus, although most studies using sonoporation to enhance therapy delivery employ long pulse lengths, the use of shorter pulse lengths may also be employed to aid in therapy delivery rates, avoid MBs aggregation and related deleterious outcomes to surrounding normal tissue [31].

The biological effects of microbubble sonoporation on the cell membrane are of particular interest to theragnostic research. Wang et al. demonstrated that sonoporation induces disruption of the cytoskeleton, in particular the alfa-tubulin arrangement, and enhances permeability of the cell membrane to MBs, a process that highly correlates with acoustic power. Furthermore, Wang et al. demonstrated that in the intracellular delivery facilitated by sonoporation, the enhancement rate of membrane permeability correlates with the disassembly of the cytoskeleton network [32,33,34].

## 3. Alternative Formulations

Despite the advantageous biological effects generated by MBs’ sonoporation, extensive research has been employed to improve its penetration into the vessel walls, for which the bubble size continues to confer a limitation. The normal endothelial tissue is able to permit diffusion of particles between about 380 and 780 nm, which limits MBs passive diffusion. Thus, the use of nanobubbles (NBs) has also been extensively studied in recent years in an effort to overcome particle size challenges and enhance drug delivery. Similar to MBs, NBs are composed of different shell and core materials and may be coupled with specific tissue ligands for targeted tissue delivery. Several studies have demonstrated the higher passive extravasation rate of NBs compared with micron-sized bubbles. These smaller contrast agents are capable of penetrating tissues more easily and preferably by passive intact extravasation, a potential advantage to therapeutic and diagnostic applications that has been extensively explored in preclinical studies [35]. These characteristics may pose a set of advantages over micron-sized bubbles such as deeper therapeutic delivery potential in NBs-loaded and diagnostic when NBs are tagged. Although initially contrast enhancement was a concern with the use of NBs, recently, phospholipid-shell formulations have demonstrated better ultrasound enhancement performance. Moreover, a higher retention time was demonstrated once tagged NBs arrived at the target tissue given its higher extravascular permeability. Sonification of the tissue of interest tends to generate coalescence of NBs into MBs, which increases its acoustic radiance enhancement properties [36].

Despite its advantages, NBs preparation still poses several challenges to its clinical application. These include the need for centrifugation prior to injection, a higher rate of impurities caused by byproducts, and the need of amphilic surfactants [37]. Initial reports demonstrated concern with NBs regarding their much lower echogenicity in ultrasound imaging compared to MBs [37,38]. Furthermore, due to the typical disorganized intra-tumoral vascular structure and consequent reduced internal blood flow, NBs’ distribution may become limited beyond the vascular endothelial wall, and there is a concern with interpretation of a potential uneven response. Despite its preclinical excitement, clinical studies using NBs have not been so fruitful, a disparity probably explained by the heterogeneity of tumoral microenvironment that influences NBs distribution and response.

Droplets and nanodroplets, also known as pulse-change emulsions, encompass a recent new category of cavitation particles being studied for diagnostic and therapeutic interventions. Liquid emulsion in droplets arose from the need for more stable cavitation agents with longer blood-half-life than MBs, since the liquid core prevents gas dissolution. Oil emulsions stabilized by surfactant coating are already in use in different clinical applications, such as aerosols and penicillin droplets. Droplets have also found use in a variety of diagnostic applications including fluorine-19 magnetic resonance imaging [39], positron emission tomography [40], and ultrasound [41] imaging. The development of triggered and controlled release of therapeutic payload was explored with the use of superheated core such as perfluorocarbons. The superheated core droplets are able to remain stable until exposed to an external stimulus, such as focused ultrasound, whereby liquid-to-gas transition takes place. After sonification, the nano-droplet vaporizes into a gas bubble in the target tissue and becomes susceptible to the same cavitation changes described for MBs earlier. The main advantages of the superheated core nano-droplet technology are the possibility of increased acoustic emissions and harmonic content in the target tissue, with longer circulation time and cell membrane diffusion capability [39,40,41].

Although promising, the use of droplets in clinical oncologic applications still has several challenges to overcome. Since the efficacy of ultrasound enhancement and tumor targeting of droplets largely depend on shell and perfluorocarbon core composition, studies are still needed to define the best materials for clinical application. The ideal threshold of vaporization while maintaining thermal stability demands a definition of a material that will remain stable at physiological temperatures and transition into a microbubble at a low vaporization threshold without damaging the surrounding normal tissue [42]. The first generation of droplets liquid emulsion is using perfluorocarbon.

## 4. Types of Shell Composition

There are two general types of bubbles known to be responsive to the sonoporation application: free bubbles and encapsulated bubbles. Free bubbles are simply cavities filled with air or other gases while encapsulated MBs consist of cavities surrounded by a capsule of variable composition. Table 1 summarizes types of carriers and their properties.

The sonoporation clinical application was first studied using free bubbles. The physical dynamics of free bubbles are described by the Rayleigh–Plesset equation and will produce, essentially, the same biophysical effects described earlier characterized by sonoporation. However, unlike encapsulated MBs, free bubbles have no boundaries, and under the biophysical effects of ultrasound, compression and decompression leads to instability and effects are poorly predictable [43].

Similar to free bubbles, encapsulated MBs are able to circulate in the blood stream until they reach the area of interest. The biological effects of MBs are highly dependent on the gas core and shell composition and respond differently according to US setting parameters. Over the years, researchers developed numerous strategies to enhance MBs stability and targeting by coating with polymers, proteins, or lipids. The engineering of different combinations of shell and gaseous core also allowed improvement of scattering effect and contrast-to-background tissue ratio [44].

Currently, phospholipid coating is the most used shell composition, since it allows for high biocompatibility, flexibility, and enhanced non-linear properties. Moreover, a phospholipid coating may be further enhanced by the addition of numerous molecules, such as polyethylene glycol (PEG), which reduces interaction with immunologic cells and allows coupling with targeting ligands, genes, or chemotherapeutics, turning them into potential therapy microcarriers [45].

As mentioned above, the cavitation behavior of MBs varies greatly between different shell compositions given their particular viscoelastic properties. For instance, phospholipidic shells are typically composed of a thinner flexible layer which allows them to oscillate at low acoustic pressures. Once rupture is reached, these lipidic MBs tend to form smaller subsets which surround the main particle. Hard shell MBs, in contrast, typically bear a thicker shell layer and thus require higher acoustic pressures to reach cavitation. Moreover, due to higher acoustic pressure, fragmentation of hard-shelled MBs results in a more aggressive fragmentation known as sonic cracking, capable of propelling the core gas a few microns away [18,46,47]. Liu et al. analyzed molecule delivery efficiency of MBs in 26 studies using different US parameters and shell compositions. The authors noted that a more efficient uptake was associated with the use of Definity contrast agent (lipid shell) compared to Optison (albumin shell) in the analyzed studies [48]. The authors also highlighted the association of temperature with higher uptake efficiency, with better results reported at 37 °C. In an effort to minimize confounding variables from different ultrasound parameters and MBs concentration from the different studies, Liu et al. reproduced an experiment with fixed US parameters and confirmed higher dextran delivery efficiency using Definity contrast agents compared to Optison at 37 or 23 °C.

**Table 1:** Types of carriers and their properties.

	Mechanism	Cavitation Threshold	Advantages	Limitations	References
Lipid shell	Cavitation, endocytosis	Low	Easy labeling and therapy loading. Low immunogenic profile	Limited loading capacity	[18,40,42]
Albumin shell	Disulfide crosslink formation and fragmentation	High	Simple formulation	Unable to bind negatively charged molecules	[46,47]
Polymer shell	Fragmentation	High	Able to accommodate hydrophobic and hydrophilic molecules	High cavitation threshold may damage surrounding normal tissue	[49]
Nanobubbles	Cavitation and aggregation	Low	Passive endothelial penetration	Low echogenicity impairs contrast and potentially tissue targeting	[35,36,37,38]
Droplets	Pulse-change emulsion	Variable	Increased half-life by avoiding immediate gas dissolution	Narrow cavitation threshold	[39,40,41]
Nanoparticles	Hyperthermia, cavitation, free radicals	Variable	Functionalization	Safety profile, variable and/or unknown toxicity	[50]

Hard shell MBs (e.g., albumin, polymer) typically require higher acoustic pressures to induce pore formation. In contrast to lipid shell MBs, the process of cavitation in hard shell MBs is known as sonic cracking, with fracturing of the capsule and subsequent release of the gas core. Ultrasound pressure thresholds for sonic cracking are reported between 400 and 1200 kPa, typically higher than the ones needed for lipid-shell cavitation. Induced cell permeability correlates with the extent of sonic cracking allowing effective large pore formation; however, normal tissue and endothelial cell viability remains a concern at higher acoustic power parameters [49].

The simplest way to load albumin-shell MBs is by incubation of viral vectors and drugs of interest. Unfortunately, this strategy is inefficient for coating non-viral gene therapy due to the negative charges on both the protein shell and the nucleic acid backbone. A second mechanism utilized to circumvent this limitation is the gene therapy crosslinking into the protein matrix at its formation stage. This has shown to effectively deliver therapy when sonification is applied by fragmentation [51,52].

## 5. Kinetics

MBs are isotonic to human plasma and can circulate through capillaries, given their small size. After intravenous injection, MBs dissolve producing remnants that are readily metabolized and cleared, minimizing risk of emboli. The biodistribution and clearance properties vary greatly between different MBs’ shell and core compositions with reported half-lives ranging from 1 min for albumin shell and air core to up to 180 min in lipid shell with N2/perfluorohexane core [53]. The short half-life of most commonly used ultrasound contrast agent MBs is caused by their temporary retention in lung, liver, and spleen along with their rapid disintegration in small vessels. The safety profile of MBs is considered good, with rare reported side effects including dizziness, erythematous rash, itching, nausea, vomiting, dyspnea, bronchospasm, hypotension, bradycardia, cutaneous rash, back pain, and clouding of consciousness [54].

## 6. Particle Formation and Labeling

One of the main advantages of MBs is the possibility to target these particles to bind specific tissues, improving focused drug delivery and the possibility of increasing dose while minimizing side effects related to systemic distribution. Targeted MBs can bind to specific receptors in a tissue of interest and allow for uptake density estimation by high intensity pulse sonification of the region. MBs may be engineered to target specific tissues by attachment of antibodies through avidin-biotin, maleimide-thiol, carboxylic acid-amine [55], or by coupling the ligand to the shell prior to particle formation [56]. Avidin-biotin ligands have been extensively used in preclinical research, since they can be easily incorporated to the shell layer and allow multiple attachments, made possible by their four avidin binding sites. A known disadvantage of this approach is the high avidin immunogenicity in humans, precluding its use in clinical trials. On the other hand, covalent bonds such as maleimide-thiol and carboxylic acid amine coupling avoid immunogenic reactions when all groups are bonded [57].

PEGylation is a well-known technique used to create a shield around particles, reducing opsonization and subsequent clearance by the reticuloendothelial system. PEG has been incorporated to therapeutics and the MBs shell to stabilize carriers against coalescence with other MBs and to increase the circulatory lifetime. PEG chain spacing and long tethered adhesion ligands also demonstrated improved performance with higher affinity for antibody adhesion [58]. One of the concerns with MBs’ targeting was that the exposure of ligands to the immunologic system could reduce systemic half-life and increase risk of immunologic reactions. A new strategy to overcome the immunogenic reaction or interaction of ligands with other tissues was demonstrated by Borden et al. by burring the ligand in the PEG brush [59]. The authors demonstrated that when the region of interest is sonificated, ultrasound radiation forces lead to unfolding of the PEG chain and exposure of the ligand to the specific target.

Recently, however, authors have raised attention to the accelerated microbubble clearance observed after multiple exposures to PEG-coated MBs [60]. The mechanism is thought to result from the production of anti-PEG antibodies, which was observed to progressively reduce target binding in ultrasound molecular studies over time. This process may limit a study’s interpretation since changes in target binding may reflect changes in MBs pharmacokinetics and not necessarily changes in tumor target expression.

Besides the construction of a tagged microbubble shell, other techniques to combine gene delivery systems and MBs include the simple physical mixture and binding of the gene to the microbubble surface by electrostatic or chemical binding.

## 7. Dual Vector Approach

As mentioned earlier, the main advantage of the use of viral vectors is their high rate of transfection compared to non-viral vectors, with the disadvantage of a high immunogenic profile. To circumvent this limitation and combine the advantages of two promising vectors, researchers developed a gene and drug theragnostic system by incorporating the viral vectors in MBs. MBs provide a clinically safe and efficient protection to target viral delivery while protecting the viral vector from inciting immunologic response, avoiding undesirable side effects and precocious inactivation. Previous work has shown successful results using ultrasound-mediated delivery of Adenoviruses and therapeutic DNA into tumor xenografts in both immune-incompetent and immune-competent animal models [61,62]. The conjugate microbubble–viral vector was obtained by simple admixture at the MBs reconstitution stage with adenovirus. After restitution, MBs’ surface binding adenovirus were effectively deactivated by complement. This is an important step to avoid immunogenicity and toxicity. MBs containing encapsulated adenovirus were collected from the complement incubation and injected in mice. The authors demonstrated a high level of site-specific transfection without expression in other organs or toxicity. Moreover, the authors demonstrated that the microbubble shell effectively protects the viral vector from inactivation by both humoral and cellular immunity response in a murine model [61].

Yoon et al. described another dual vector approach utilizing MB–liposome complex. The conjugate was prepared utilizing thiol-active MBs derived from DSPE-PEG-PDP shaking for 2 h with thiolated liposome. The conjugate had a surface amine functional group and allowed for specific ligand targeting [63]. Park et al. further established a microbubble–liposome complex conjugate coupled with target ligands to enhance delivery of chemotherapy to brain tumors. For this study, interleukin-4 receptor targeting peptides were conjugated to MB-doxorubicin-loaded liposomes to improve drug delivery to brain tumors compared to non-targeted therapy. The authors demonstrated 30% decrease in viability with ultrasound-guided conjugate site delivery compared to non-targeted particles [64].

The MB component in both approaches is used to generate high echogenic signals, allowing for enhanced real-time tissue characterization. While liposomes are effective versatile therapeutic carriers and adenoviral vectors provide effective gene transfection, when combined with specific delivery through MBs sonoporation effects further enhance intracellular delivery and improve safety profile.

## 8. Therapeutic Applications

Su et al. reported on a new approach for remote high intensity ultrasound-guided delivery of poly-(lactic-co-glycolic acid) (PLGA) microparticles to promote prolonged sustained targeted therapy. The authors observed a sustained release of therapeutics for up to two weeks after ultrasound-guided implantation of microparticles with increased therapeutic-load compared to controls, highlighting the potential of this technique in the enhancement of high intensity ultrasound applications in tumor tissue [65].

Do et al. applied a similar technique using high intensity ultrasound enhancement of intratumorally-delivered PLGA microspheres loaded with doxorubicin in melanoma tumors demonstrating the synergistic effect of direct and indirect ultrasound of microspheres loaded with doxorubicin with increased half-life in treated mice compared to untreated and single treatments [66]. The authors relied on previously reported therapeutic effects of ultrasound and hypothesized that direct and indirect ultrasound biological effects over the insonated tissue could increase tumor response to non-acoustically active microspheres. The mentioned direct effects include varying degrees of pore formation either leading to lethal cell membrane damage, or reversible pore formation resulting in facilitated microsphere delivery. Additional indirect effects included inertial cavitation to neighboring free air bubbles and microsphere collision, promoting enhanced local release of doxorubicin, as previously demonstrated by Jang et al. [67]. To optimize the imaging enhancement of the system, Chen et al. explored in a recent publication a new formulation addressing the issue. The authors combined the known advantages of lipid coating MBs in a lipid/PLGA hybrid carrier capable of theragnostic application with significantly enhanced doxorubicin delivery and acoustic response in vivo and in vitro [68].

Solid nanoparticles have been studied for ultrasound imaging and therapeutic applications. Chen et al. highlighted the multifunctionality of silica nanoparticles and demonstrated the potential for stem cell targeting by incorporation of bis(triethoxysilyl) ethane and bis(3-trimethoxysilyl-propyl) amine, which created a concave mesoporus structure similar to exosome extracellular vesicle. The new morphology was shown to increase ultrasound contrast and affinity to stem cells in vivo and in vitro [69]. Although the authors described the use for imaging purposes, there is growing interest in stem cells targeting for cancer therapy [70].

Rinaldi et al. recently reported on the successful use of ultrasound-induced sonoporation of commercially available MBs (Sonovue®) as vectors for expression induction of silenced genes such as *TRAIL* and *p53* in liver cancer cells. Additionally, the authors demonstrated increased apoptotic effect by combining epigenetic treatment such as histone deacetylase inhibitor to enhance the pro apoptotic effect of *TRAIL* gene expression [71].

Innovative uses of ultrasound contrast agents have been recently published, testing the use of ultrasound in once thought unreachable tissues such as the peritoneum and the ovaries. Nishimura et al. reported on the effects of site-specific ultrasound-induced sonoporation of nanobubbles and the concomitant administration of naked pDNA for treatment of peritoneal fibrosis. Surprisingly, a 10-fold higher transgene expression to peritoneal mesothelial cells was observed compared to injection alone with no toxicity to the adjacent liver [72]. The authors highlight the need of a laparoscopic intraperitoneal ultrasound probe capable of safe intra-abdominal use to achieve site-specific sonoporation which has been previously developed [73,74].

Along those lines, the use of ultrasound contrast agents in ovarian cancer imaging have been described using CA-125-targeted echogenic lipid and surfactant-stabilized nanobubbles in vitro and in vivo with significant enhanced tumor accumulation, high signal intensity, and slower wash out rates in CA-125 positive (OVCAR-3) compared to CA-125 negative cell lines (SKOV-3) [75]. Li et al. compared non-targeted lipid microbubbles and luteinizing hormone releasing hormone analogue-targeted lipid microbubble efficacy in vitro transfection of OVCAR-3 cell lines with the Von Hippel Lindau (VHL) gene. The authors demonstrated enhanced VHL expression using ultrasound microbubble mediated delivery with greater regulatory effect compared to injection alone [76].

In contrast to the superficial tissues and the abdominal cavity, chemotherapy delivery for treatment of brain tumors is largely limited by the blood–brain barrier (BBB). To achieve the required therapeutic concentration in brain tumors the drug often reaches cytotoxic concentration systemically. Researchers have been exploring the use of sonoporation to induce transient increased permeability of the BBB with and without drug delivery. Liu et al. investigated the feasibility and effectiveness of high intensity focused ultrasound combined with MBs to transiently disrupt the BBB and significantly enhance the delivery of 1,3-bis(2-chloroethyl)-1-nitrosourea to glioblastoma cells in rats under magnetic resonance imaging (MRI) monitoring. The authors reported a 340% increase in BCNU concentration in sonicated brains and a two-fold increase in the tumor compared to drug injection alone at 0.62 MPa. Given that the heating effects from focused high intensity ultrasound could potentially damage the surrounding normal cells, the authors tested the safety of a range of acoustic pressure from 0.48 to 1.35 MPa. A significant increase in brain enhancement was obtained at 0.62 MPa without signs of normal tissue damage, while at 0.98 MPa intracranial hemorrhage was reported without significant further increase in BBB permeability [77]. Recently, Liu et al. explored the advantages of the transient BBB opening using the same technique to overcome the vascular normalization phenomena in anti-vascular endothelial growth factor (VEGF) therapy for brain tumors. The authors again reported encouraging results with a 57-fold increase in drug concentration, successfully overcoming the vascular normalization challenge [78].

Similar to the BBB, the effects of ultrasound microbubble sonoporation have been explored to enhance permeability of the round membrane and improve drug delivery to the inner ear. The authors explored the efficiency of permeability induction by ultrasound and MBs and aided delivery of nanoparticles in a combined setting. By applying MB-induced sonoporation, the authors achieved transient reversible disruption of the epithelial tight junctions of the round window outer surface membrane, successfully improving site specific drug delivery [79]. Table 2 summarizes clinical trials using ultrasound-mediated therapeutic applications.

## 9. Conclusions and Future Perspectives

The therapeutic and diagnostic application of ultrasound guided MBs and particle sonoporation has drastically evolved in recent years with the use of computational high-speed imaging techniques that have provided a better understanding of the mechanics involved in sonoporation and the development of new, more versatile agents. With concurrent growing interest in individualized targeted therapy with minimized side effects, the use of ultrasound-mediated local delivery agents has been explored with promising results, including but not limited to cross-linking with chemotherapeutics, gene therapy, and sonodynamics.

Nonetheless, over the years, the initial excitement with the new gene therapy field faced a series of challenges and drawbacks. Although much effort has been made to advance knowledge and clinical applicability, many questions remain unsolved and application in clinical trials is only modest. Of the many challenges encountered, the need for vectors that could effectively and safely deliver drugs or transfect genetic material remains a main area of study and development. The emergence of ultrasound contrast agents (MBs) brought a lot of excitement with promising applicability in therapy and diagnosis.

Although promising, there are still many challenges to overcome. The major limitation to date consists of the rate-limitation imposed by the endothelium. Furthermore, most of the drug delivery is given primarily in the surroundings of the subluminal intima of the vasculature. New developments using alternative formulations using droplets and nanobubbles have explored this limitation as mentioned previously; however, they still lack control over the delivery beyond the endothelium. New approaches are needed that allow exact quantification of drug load to targeted tissues.

The number of variables that come into play to determine the type of result from cells, MBs, and ultrasound interactions were highlighted in this paper and raises attention to the complexity of these phenomena. Notwithstanding the progressive understanding of MBs biomechanics, standardization of ultrasound parameters and microbubble properties remain a major challenge to proper safety determination and clinical translation.

## Figures and Tables

**Table 2:** Summary of clinical trials using ultrasound-mediated therapeutics for cancer applications.

Target	Function	Therapeutic	Carrier	Identifier
Liver metastases from gastrointestinal system	Drug carrier	Platinum and Gencitabim	Microbubble	NCT02233205
Liver metastases from colorectal cancer	Drug delivery enhancer	Monoclonal antibody chemotherapy	Microbubble (Sonovue ^®^)	NCT03458975
Hepatocellular carcinoma	Drug delivery enhancer	Yttrium-90 microspheres	Perflutren protein-type A microspheres	NCT03199274
Liver tumor	Drug delivery	Doxorrubicin	Lyso-thermosesnsitive liposoma	NCT02181075
Pancreatic cancer	Drug delivery enhancer	Multiple chemotherapy	Microbubble	NCT04821284
Glioblastoma	BBB permeability	Bevacizumab	Microbubble (Sonovue ^®^)	NCT04446416
Glioblastoma	BBB permeability	Carboplatin	Microbubble	NCT04417088
Diffuse Midline Glioma	BBB permeability	Panobinostat	Microbubble	NCT04804709
Glioblastoma	BBB permeability	Abraxane	Microbubble	NCT04528680
Breast cancer	Drug delivery enhancer	Neoadjuvant chemotherapy	Microbubble	NCT03385200
Pediatric refractory solid tumors	Drug delivery	Doxorrubicin	Lyso-thermosensitive liposoma	NCT02536183
